# Obesity as a Risk Factor for Carpal Tunnel Syndrome Independent of Diabetes Mellitus: A Nationwide Study

**DOI:** 10.1016/j.jhsg.2025.01.016

**Published:** 2025-03-08

**Authors:** Gregory R. Vance, Katherine Benedict, Clay B. Thames, Bradley F. Hathaway, Evan C. Bowen, Marc E. Walker

**Affiliations:** ∗ Division of Plastic and Reconstructive Surgery, University of Mississippi Medical Center, Jackson, MS; † Department of Plastic Surgery, MD Anderson Cancer Center, Houston, TX

**Keywords:** Carpal, Diabetes, Obesity, Syndrome, Tunnel

## Abstract

**Purpose:**

Although the relationship between diabetes mellitus (DM) and carpal tunnel syndrome (CTS) is well documented because of diabetic neuropathic complications, a relationship specific to CTS and obesity has not yet been identified on a population level. The current study seeks to compare CTS prevalence between obese and nonobese patients and examine relationships among obesity, DM, and CTS.

**Methods:**

Data used in this study came from Epic Cosmos, a community collaboration of health systems representing over 227,000,000 patient records from over 1,301 hospitals and 28,600 clinics. All patients at least 18 years of age with an encounter between December 2013 and December 2023 were included and grouped based on presence or absence of International Classification of Diseases-10 codes for CTS, obesity, and DM as well as Current Procedural Terminology codes for EMG and nerve conduction studies. Here, 99% confidence intervals were recorded, and odds ratios (OR) were calculated for group comparison using *P* < .01 for significance.

**Results:**

All adult patients with a documented obesity diagnosis showed a near six-fold increase in CTS prevalence compared with nonobese adult patients. When excluding those with a documented DM diagnosis, this relationship was largely maintained with a five-fold increased prevalence in obese adults without DM compared with nonobese adults without DM. Additionally, obese adults without DM had a higher rate of CTS compared to nonobese, diabetic adults.

**Conclusions:**

Although DM patients are classically associated with CTS presentation, obesity even without DM may possess a similar or more powerful relationship. Additionally, comorbidity of obesity and DM suggests an additive effect between the two diagnoses on increased CTS prevalence. However, further research must be performed to evaluate the anatomic and pathologic mechanism of this relationship.

**Type of study/level of evidence:**

Differential Diagnosis/Symptom Prevalence Study, Level 2b.

Both diabetes mellitus (DM) and carpal tunnel syndrome (CTS) are increasingly common, especially with the rise of sedentary lifestyles and progressively advancing age.^1^ Although the rate of CTS in the general population ranges from 1% to 5%, prior research has suggested a complex interplay between the development of DM and CTS, regardless of type 1 diabetes mellitus (T1DM) or type 2 diabetes mellitus (T2DM) designation.^1^, ^2^, ^3^ In addition, diabetic peripheral neuropathy is a well-established complication of diabetes and often exhibits wide-ranging overlap with CTS, thereby complicating the exploration of etiology between these conditions.^2^ As the obesity epidemic remains a major global health concern and is well-connected to DM pathology, a study highlighting the specific relationship between CTS and obesity holds considerable promise and could guide the development of medical prevention and treatment strategies.

DM and obesity have been independently associated with CTS in the context of genetics.^4^ Although mechanisms involved in the development of CTS from increased adiposity are not fully understood, prior studies suggest direct implications and connections to metabolic syndromes that increase risk.^4^ Considering the associations of smoking, thyroid diseases, and repetitive maneuvers is also recommended when evaluating the multifactorial components associated with CTS.^5^^,^^6^ In contrast to this genetic approach, the current study seeks to compare CTS prevalence among obese and nonobese patients through a phenotypic perspective, as there is currently a lack of literature providing evidence for this connection.

In addition to evaluating the independent impact of obesity on CTS, the study aims to explore the interconnected relationships among obesity, DM, and CTS. Furthermore, it aims to evaluate obesity as a risk factor for CTS independent of DM pathology, also prompting the question of whether comorbidities of obesity and diabetes have an additive effect on CTS prevalence. Extending the scope of the current literature to the combination of obesity, diabetes, and CTS creates a unique approach that helps shed light on the complex relationship of the diseases.

On a socioeconomic level, CTS has also been related to occupations associated with consistent or repetitive hand maneuvers, especially those against resistance.^7^ Notably, CTS is responsible for a significant portion of work-related disability, thus negatively affecting patients partly through the accumulation of substantial health care costs.^2^^,^^4^ Therefore, establishing a relationship among obesity, DM, and CTS and further promoting obesity prevention may help reduce high health care expenditures associated with CTS-related occupational disability.

Additionally, the cost of health care associated with managing each of these conditions individually dwindles in comparison to the expenses incurred when treating patients who were experiencing a combination of these conditions.^8^ Expenses related to managing comorbidities and complications could be mitigated by exploiting the interplay between these conditions. An integrated treatment approach that addresses both obesity and DM could lead to an even greater potential decrease in CTS incidence and health care expenditures.^9^^,^^10^

## Materials and Methods

Data used in this study came from Epic Cosmos, a community collaboration of health systems representing over 227,000,000 patient records from over 1,301 hospitals and 28,600 clinics. Because the Epic Cosmos database includes intrinsically deidentified data, informed consent and reviewal by an institutional review board was not necessary for the purposes of this study. All patients at least 18 years of age with an encounter between December 7, 2013, and December 6, 2023 (N = 188,112,962), were included and grouped based on presence or absence of documented International Classification of Diseases-10 (ICD-10) codes for obesity, body mass index (BMI) ranges, and DM. Analyses were also performed specific to T1DM and type 1 diabetes mellitus (T2DM). Further analyses within these groups were conducted to specify by patients with DM with a recorded hemoglobin A1C (HbA1C) >6.5%. The specific ICD-10 codes used and respective population sizes are shown in Table 1. Additionally, patients with a recorded diagnosis of obesity with recorded prescription of orlistat, lorcaserin, phentermine/topiramate, naltrexone/bupropion, or liraglutide were compared to those who were not prescribed pharmaceutical treatment.

**Table 1:** ICD-10 and Current Procedural Terminology Codes Used for Studied Diagnoses, BMI Ranges, and Procedures

Diagnosis/Procedure	ICD-10 or Current Procedural Terminology Code(s)	N
Carpal tunnel syndrome	G56.0, G56.00, G56.01, G56.02, G56.03	3,426,821
Body mass index		
<19.9	Z68.1	1,937,281
20.0–29.9	Z68.2, Z68.20, Z68.21, Z68.22, Z68.23, Z68.24, Z68.25, Z68.26, Z68.27, Z68.28, Z68.29	165,403,966
30.0–39.9	Z68.3, Z68.30, Z68.31, Z68.32, Z68.33, Z68.34, Z68.35, Z68.36, Z68.37, Z68.38, Z68.39	13,542,382
40.0–49.9	Z68.41, Z68.42	3,316,957
50.0–59.9	Z68.43	563,017
60.0–69.9	Z68.44	99,203
>70.0	Z68.45	38,837
Obesity	E66, E66.∗, E66.0, E66.01, E66.09, E66.1, E66.2, E66.3, E66.8, E66.9	26,702,609
Diabetes mellitus	E08, E09, E10, E11, E13	18,462,700
Nerve conduction studies	95907, 95908, 95909, 95910, 95911, 95912, 95913	---
EMG	95860, 95861, 95863, 95864, 95885, 95886, 95870	---

Prevalence of comorbid CTS and 99% CIs were measured using the SlicerDicer feature within Epic Cosmos. These measurements were repeated to include only patients with a recorded EMG and nerve conduction studies (NCS) within the found CTS prevalence, in order to specify by EMG/NCS-confirmed CTS. For each analysis, the “Population” within SlicerDicer was determined by patients with documented ICD-10 diagnosis of obesity, DM, or the BMI ranges listed above and were repeated with recorded HbA1C >6.5% as applicable. The “Percent with Selected Value” function was then used to determine documented CTS prevalence by ICD-10 code in each of these groups, and this function was used again for each population group to specify by EMG/NCS-confirmed CTS diagnosis using Current Procedural Terminology codes, which can be found in Table 1. 99% CIs were generated within SlicerDicer for each prevalence value. Using MedCalc, odds ratios with respective *P* values for CTS diagnosis were calculated for obese versus nonobese adults and among BMI ranges in increments of 10.0. This population was then stratified to compare obese versus nonobese adults in those both with and without DM. Significance was determined by *P* < .01. The Strengthening the Reporting of Observational Studies in Epidemiology (STROBE) guideline checklist was used to optimize the strength of data reporting in this study.

## Results

### Relationship among obesity, DM, and CTS

Diagnosis of obesity was associated with a significantly increased prevalence of CTS in all studied patients (*P* < .001), in patients with DM (*P* < .001), and in patients without DM (*P* < .001). In nondiabetic patients, this prevalence was increased by almost five-fold. This relationship was consistent when including a recorded HbA1C greater than 6.5% (*P* < .001), specifying by EMG/NCS-confirmed CTS (*P* < .001), and in analyses specific to T1DM (*P* < .001) and T2DM (*P* < .001). Additionally, nondiabetic patients with obesity exhibited a significantly increased prevalence of CTS diagnosis compared with nonobese diabetic patients (*P* < .001). This relationship was maintained in all analyses except in patients with T1DM with a confirmed HbA1C greater than 6.5% and specified by EMG/NCS-confirmed CTS (*P* = .053). Our results were consistent with the hypothesis of obesity as an independent risk factor for CTS. These results are further represented in Table 2, Table 3, Table 4, and 5. Analyses comparing T1DM and T2DM supported a significantly increased CTS prevalence in obese patients with T1DM (*P* < .001), as shown in Table 6. Additionally, patients requiring medical treatment for obesity showed a significant two-fold increase in CTS prevalence compared to those without pharmaceutical intervention (*P* < .001), as demonstrated in Table 7.

**Table 2:** Comparing CTS Prevalence in Obese Adults

Patient Group	CTS Prevalence	Odds Ratio	*P* Value
No recorded EMG/NCS			
Obese adults	5.9% ± 0.01%		
Nonobese adults	1.1% ± 0.00%	5.41	<.001∗
Recorded EMG/NCS			
Obese adults	1.8% ± 0.01%		
Nonobese adults	0.3% ± 0.00%	5.36	<.001∗

∗ *P* < .01, indicating statistical significance.

**Table 3:** Comparing Prevalence of CTS Based on Diagnosis of Obesity and DM

Control Group	Comparison Group	CTS	OR	*P*
No recorded EMG/NCS				
Obese adults with DM		7.9% ± 0.03%		
	Obese adults without DM	5.0% ± 0.01%	1.62	<.001∗
	Nonobese adults with DM	3.1% ± 0.01%	2.67	<.001∗
	Nonobese adults without DM	1.0% ± 0.00%	8.44	<.001∗
Obese adults without DM		5.0% ± 0.01%		
	Nonobese adults with DM	3.1% ± 0.01%	1.65	<.001∗
	Nonobese adults without DM	1.0% ± 0.00%	5.21	<.001∗
Obese adults with DM and HbA1C > 6.5%		9.1% ± 0.03%		
	Obese adults without DM	5.0% ± 0.01%	1.89	<.001∗
	Nonobese adults with DM and HbA1C > 6.5%	4.4% ± 0.02%	2.20	<.001∗
	Nonobese adults without DM	1.0% ± 0.00%	9.81	<.001∗
Obese adults without DM		5.0% ± 0.01%		
	Nonobese adults with DM and HbA1C > 6.5%	4.4% ± 0.02%	1.17	<.001∗
Recorded EMG/NCS				
Obese adults with DM		2.6% ± 0.01%		
	Obese adults without DM	1.4% ± 0.01%	1.81	<.001∗
	Nonobese adults with DM	0.9% ± 0.01%	2.98	<.001∗
	Nonobese adults without DM	0.3% ± 0.00%	8.98	<.001∗
Obese adults without DM		1.4% ± 0.01%		
	Nonobese adults with DM	0.9% ± 0.01%	1.64	<.001∗
	Nonobese adults without DM	0.3% ± 0.00%	4.95	<.001∗
Obese adults with DM and HbA1C > 6.5%		3.0% ± 0.02%		
	Obese adults without DM	1.4% ± 0.01%	2.11	<.001∗
	Nonobese adults with DM and HbA1C > 6.5%	1.2% ± 0.01%	2.53	<.001∗
	Nonobese adults without DM	0.3% ± 0.00%	10.42	<.001∗
Obese adults without DM		1.4% ± 0.01%		
	Nonobese adults with DM and HbA1C > 6.5%	1.2% ± 0.01%	1.20	<.001∗

∗ *P* < .01, indicating statistical significance.

**Table 4:** Comparing Prevalence of CTS Based on Diagnosis of Obesity and T1DM†

Control Group	Comparison Group	CTS	OR	*P*
No recorded EMG/NCS				
Obese adults with T1DM		10.4% ± 0.01%		
	Obese adults without DM	5.0% ± 0.01%	2.19	<.001∗
	Nonobese adults with T1DM	4.1% ± 0.05%	2.75	<.001∗
	Nonobese adults without DM	1.0% ± 0.00%	11.38	<.001∗
Obese adults without DM		5.0% ± 0.01%		
	Nonobese adults with T1DM	4.1% ± 0.05%	1.26	<.001∗
Obese adults with T1DM and HbA1C > 6.5%		11.2% ± 0.11%		
	Obese adults without DM	5.0% ± 0.01%	2.36	<.001∗
	Nonobese adults with T1DM and HbA1C > 6.5%	4.8% ± 0.07%	2.48	<.001∗
	Nonobese adults without DM	1.0% ± 0.00%	12.31	<.001∗
Obese adults without DM		5.0% ± 0.01%		
	Nonobese adults with T1DM and HbA1C > 6.5%	4.8% ± 0.07%	1.05	<.001∗
Recorded EMG/NCS				
Obese adults with T1DM		3.7% ± 0.06%		
	Obese adults without DM	1.4% ± 0.01%	2.65	<.001∗
	Nonobese adults with T1DM	1.2% ± 0.03%	3.26	<.001∗
	Nonobese adults without DM	0.3% ± 0.00%	13.11	<.001∗
Obese adults without DM		1.4% ± 0.01%		
	Nonobese adults with T1DM	1.2% ± 0.03%	1.23	<.001∗
Obese adults with T1DM and HbA1C > 6.5%		4.1% ± 0.07%		
	Obese adults without DM	1.4% ± 0.01%	2.94	<.001∗
	Nonobese adults with T1DM and HbA1C > 6.5%	1.5% ± 0.04%	2.89	<.001∗
	Nonobese adults without DM	0.3% ± 0.00%	14.54	<.001∗
Obese adults without DM		1.4% ± 0.01%		
	Nonobese adults with T1DM and HbA1C > 6.5%	1.5% ± 0.04%	0.98	.053

∗ *P* < .01, indicating statistical significance.

**Table 5:** Comparing Prevalence of CTS Based on Diagnosis of Obesity and T2DM

Control Group	Comparison Group	CTS	OR	*P*
No Recorded EMG/NCS				
Obese adults with T2DM		8.2% ± 0.02%		
	Obese adults without DM	5.0% ± 0.01%	1.69	<.001∗
	Nonobese adults with T2DM	3.4% ± 0.01%	2.56	<.001∗
	Nonobese adults without DM	1.0% ± 0.00%	8.78	<.001∗
Obese adults without DM		5.0% ± 0.01%		
	Nonobese adults with T2DM	3.4% ± 0.01%	1.52	<.001∗
Obese adults with T2DM and HbA1C > 6.5%		9.1% ± 0.03%		
	Obese adults without DM	5.0% ± 0.01%	1.89	<.001∗
	Nonobese adults with T2DM and HbA1C > 6.5%	4.4% ± 0.02%	2.18	<.001∗
	Nonobese adults without DM	1.0% ± 0.00%	9.84	<.001∗
Obese adults without DM		5.0% ± 0.01%		
	Nonobese adults with T2DM and HbA1C > 6.5%	4.4% ± 0.02%	1.15	<.001∗
Recorded EMG/NCS				
Obese adults with T2DM		2.6% ± 0.01%		
	Obese adults without DM	1.4% ± 0.01%	1.81	<.001∗
	Nonobese adults with T2DM	0.9% ± 0.01%	2.96	<.001∗
	Nonobese adults without DM	0.3% ± 0.00%	8.97	<.001∗
Obese adults without DM		1.4% ± 0.01%		
	Nonobese adults with T2DM	0.9% ± 0.01%	1.63	<.001∗
Obese adults with T2DM and HbA1C > 6.5%		3.0% ± 0.02%		
	Obese adults without DM	1.4% ± 0.01%	2.11	<.001∗
	Nonobese adults with T2DM and HbA1C > 6.5%	1.2% ± 0.01%	2.50	<.001∗
	Nonobese adults without DM	0.3% ± 0.00%	10.46	<.001∗
Obese adults without DM		1.4% ± 0.01%		
	Nonobese adults with T2DM and HbA1C > 6.5%	1.2% ± 0.01%	1.18	<.001∗

∗ *P* < .01, indicating statistical significance.

**Table 6:** Comparing CTS Prevalence Between T1DM and T2DM

Patient Group	CTS Prevalence	Odds Ratio	*P* Value
No Recorded EMG/NCS			
Obese with T1DM	10.4% ± 0.10%		
Obese with T2DM	8.2% ± 0.02%	1.30	<.001∗
Obese with T1DM and HbA1C > 6.5%	11.2% ± 0.11%		
Obese with T2DM and HbA1C > 6.5%	9.1% ± 0.03%	1.25	<.001∗
Recorded EMG/NCS			
Obese with T1DM	3.7% ± 0.06%		
Obese with T2DM	2.6% ± 0.01%	1.46	<.001∗
Obese with T1DM and HbA1C > 6.5%	4.1% ± 0.07%		
Obese with T2DM and HbA1C > 6.5%	3.0% ± 0.02%	1.39	<.001∗

∗ *P* < .01, indicating statistical significance.

**Table 7:** Comparing CTS Prevalence in Obese Patients Based on Pharmaceutical Treatment With and Without Recorded EMG/NCS

Patient Group	CTS Prevalence	Odds Ratio	*P* Value
No Recorded EMG/NCS			
Obese adults requiring treatment	11.4% ± 0.07%		
Obese adults without treatment	6.0% ± 0.01%	2.03	<.001∗
Recorded EMG/NCS			
Obese adults requiring treatment	4.0% ± 0.04%		
Obese adults without treatment	1.7% ± 0.01%	2.42	<.001∗

∗ *P* < .01, indicating statistical significance.

### CTS prevalence related to BMI

When comparing prevalence of CTS based on BMI range, there was a significantly higher prevalence in CTS diagnosis in those with a BMI greater than 30.0 when compared to those under 30.0 (*P* < .001), in accordance with the hypothesis that obesity is a risk factor for CTS. However, when split by 10.0 BMI increments, this prevalence significantly decreased with increasing BMI values after the 30.0–39.9 group. When recorded EMG/NCS was included, prevalence increased with BMI until the 50.0–59.9 group, after which a similar decline in CTS prevalence was found. These results are shown in Table 8.

**Table 8:** Comparing CTS Prevalence Among Varying BMI Range

Control BMI (kg/m²)	Comparison BMI (kg/m²)	CTS Prevalence	Odds Ratio	*P* Value
No recorded EMG/NCS				
>70		3.4% ± 0.24%		
	60.0–69.9	3.6% ± 0.15%	0.94	0.029
	50.0–59.9	4.6% ± 0.07%	0.72	<.001∗
	40.0–49.9	5.5% ± 0.03%	0.61	<.001∗
	30.0–39.9	6.0% ± 0.02%	0.55	<.001∗
	20.0–29.9	1.2% ± 0.00%	2.80	<.001∗
	<19.9	2.7% ± 0.03%	1.25	<.001∗
60.0–69.9		3.6% ± 0.15%		
	50.0–59.9	4.6% ± 0.07%	0.77	<.001∗
	40.0–49.9	5.5% ± 0.03%	0.65	<.001∗
	30.0–39.9	6.0% ± 0.02%	0.59	<.001∗
	20.0–29.9	1.2% ± 0.00%	2.80	<.001∗
	<19.9	2.7% ± 0.03%	1.33	<.001∗
50.0–59.9		4.6% ± 0.07%		
	40.0–49.9	5.5% ± 0.03%	0.84	<.001∗
	30.0–39.9	6.0% ± 0.02%	0.77	<.001∗
	20.0–29.9	1.2% ± 0.00%	3.88	<.001∗
	<19.9	2.7% ± 0.03%	1.73	<.001∗
40.0–49.9		5.5% ± 0.03%		
	30.0–39.9	6.0% ± 0.02%	0.91	<.001∗
	20.0–29.9	1.2% ± 0.00%	4.61	<.001∗
	<19.9	2.7% ± 0.03%	2.06	<.001∗
30.0–39.9		6.0% ± 0.02%		
	20.0–29.9	1.2% ± 0.00%	5.06	<.001∗
	<19.9	2.7% ± 0.03%	2.26	<.001∗
20.0–29.9		1.2% ± 0.00%		
	<19.9	2.7% ± 0.03%	0.45	<.001∗
Recorded EMG/NCS				
>70		2.3% ± 0.10%		
	60.0–69.9	2.5% ± 0.06%	0.94	<.001∗
	50.0–59.9	2.6% ± 0.03%	0.89	<.001∗
	40.0–49.9	2.4% ± 0.01%	0.94	<.001∗
	30.0–39.9	2.1% ± 0.01%	1.09	<.001∗
	20.0–29.9	1.4% ± 0.01%	1.61	<.001∗
	<19.9	1.0% ± 0.02%	2.33	<.001∗
60.0–69.9		2.5% ± 0.06%		
	50.0–59.9	2.6% ± 0.03%	0.95	<.001∗
	40.0–49.9	2.4% ± 0.01%	1.00	.387
	30.0–39.9	2.1% ± 0.01%	1.17	<.001∗
	20.0–29.9	1.4% ± 0.01%	1.72	<.001∗
	<19.9	1.0% ± 0.02%	2.49	<.001∗
50.0–59.9		2.6% ± 0.03%		
	40.0–49.9	2.4% ± 0.01%	1.05	<.001∗
	30.0–39.9	2.1% ± 0.01%	1.22	<.001∗
	20.0–29.9	1.4% ± 0.01%	1.80	<.001∗
	<19.9	1.0% ± 0.02%	2.62	<.001∗
40.0–49.9		2.4% ± 0.01%		
	30.0–39.9	2.1% ± 0.01%	1.16	<.001∗
	20.0–29.9	1.4% ± 0.01%	1.71	<.001∗
	<19.9	1.0% ± 0.02%	2.48	<.001∗
30.0–39.9		2.1% ± 0.01%		
	20.0–29.9	1.4% ± 0.01%	1.48	<.001∗
	<19.9	1.0% ± 0.02%	2.14	<.001∗
20.0–29.9		1.4% ± 0.01%		
	<19.9	1.0% ± 0.02%	1.45	<.001∗

∗ *P* < .01, indicating statistical significance.

## Discussion

The purpose of this study was to explore the association among DM, obesity, and carpal tunnel syndrome (CTS) with the aim of understanding the association between these conditions. Our findings were consistent with previous studies which also sought to highlight the correlation between these variables.^4^^,^^11^, ^12^, ^13^ However, our study differs because of our sample size which was drawn from the nationwide Epic Cosmos database. This data set determined that CTS is more prevalent among obese patients than nonobese patients, but prevalence decreased in increasing increments of BMI after the 30.0–39.9 range group. This expands the breadth of information on CTS, obesity, and BMI previously described in prior retrospective studies, implying that BMI alone does not have a direct correlation to CTS prevalence.^14^^,^^15^

### Association between obesity and CTS

Our analysis revealed a positive association between obesity and CTS. Additionally, obese patients without DM were significantly more likely to have a documented CTS diagnosis with and without a recorded EMG/NCS than nonobese diabetic patients. Additionally, patients that required pharmaceutical treatment for obesity were more likely to have a recorded CTS diagnosis both with and without a recorded EMG/NCS than obese patients who did not require treatment. This implies that obesity alone may even be a stronger independent risk factor for CTS than isolated DM. One plausible anatomical explanation for this relationship could be increased pressure and compression of the median nerve because of excess adipose tissue, contributing to CTS symptoms.^16^ However, in this case, increasing increments of BMI would be expected to result in increased prevalence of CTS, contrary to the results of the current study. Further clinical and anatomical research is needed to determine the exact etiology of this relationship. Undoubtedly, the current findings reinforce the importance of managing obesity in CTS risk reduction.

### Association between diabetes and CTS

Similarly, this study showed that a diagnosis of DM was positively associated with CTS diagnosis, both with and without a documented EMG/NCS. These results are consistent with previous findings suggesting that diabetic neuropathy and other diabetes-related factors contribute to the development of CTS.^4^^,^^6^^,^^17^ This was especially noted among patients with T1DM, who showed significantly increased CTS prevalence compared to those with T2DM. Additional findings included that both obese and nonobese patients with DM were significantly more likely to have a documented CTS diagnosis than their nondiabetic counterparts, supporting that a diagnosis of DM is correlated with a diagnosis of CTS independent of obesity.

### Interaction of obesity and diabetes in relation to CTS

When examining the combined effect of obesity and DM on CTS, individuals who were both obese and diabetic had a significantly higher prevalence of CTS compared to those who were either diabetic or obese in isolation. Specifically, obese patients without DM were significantly more likely to have a documented CTS diagnosis than nonobese patients with DM, indicating that obesity may be a greater risk factor for CTS than DM. The only comparison that did not support this notion was between obese adults without DM versus patients with T1DM and a recorded HbA1C greater than 6.5% with a recorded EMG/NCS. Overall, this aligns with prior literature indicating that obesity and diabetes can have compounding effects on various health outcomes.^16^^,^^18^

### Major limitations

Despite the valuable insights obtained from this study, there are notable limitations. First, the cross-sectional nature of the data prevents establishment of a sequential or causal relationship among obesity, diabetes, and CTS. A future longitudinal study could be performed to demonstrate how these conditions interact over time and the potential impact of these conditions on time of CTS onset. Additionally, because ICD-10 codes were used as the criterion for diagnosis of a patient with DM, obesity, and CTS, errors or discrepancies in these codes could lead to misclassification, as coding practices and diagnostic methods vary among health systems. This study also fails to address other CTS risk factors, such as socioeconomic differences, occupational discrepancies, hypothyroidism, and pregnancy, which might have confounding effects on the data if found to be in higher proportion in obese or diabetic individuals.^5^ Finally, despite our extensive data set, all demographic variations might not be captured by this study, potentially limiting the generalizability of the findings. Further, intrinsic selection bias is likely present in the generated population, as patients afflicted with obesity, DM, and CTS who have not visited a health care center within the Epic Cosmos database were not included in this study. Future research should aim to address these limitations to strengthen the evidence for the observed associations.

## Conflicts of Interest

No benefits in any form have been received or will be received related directly to this article.
